# Jk(a) (Kidd-A) Variant in a Sickle Cell Disease Patient

**DOI:** 10.7759/cureus.49451

**Published:** 2023-11-26

**Authors:** Mohammad Barouqa, Xuebao Zhang, Ryan Walde, Zan Ahmed, Rasha Mohammed

**Affiliations:** 1 Transfusion Medicine, University of South Alabama College of Medicine, Mobile, USA; 2 Pathology and Laboratory Medicine, University of South Alabama, Mobile, USA; 3 Pathology and Laboratory Medicine, University of South Alabama Health Hospital, Mobile, USA

**Keywords:** red blood cell exchange, alloimmunization, sickle cell disease: scd, packed red blood cell transfusion, sickle cell disease complications

## Abstract

Sickle cell disease (SCD) is a chronic and prevalent hemoglobin disorder with various manifestations and complications depending on the organs involved. Red cell transfusion either simple or exchange is crucial due to its prophylactic and therapeutic roles. We present a case showing serologic discrepancy between the red cells phenotype and the developed alloantibodies to emphasize the crucial role of molecular testing in SCD patients requiring chronic blood transfusion.

## Introduction

Sickle cell disease (SCD) is an inherited, chronic, and debilitating disease caused by a single point mutation at position 6 in the *HBB* gene, which encodes the β subunit of hemoglobin. The disease is remarkably prevalent in areas with epidemic malaria infection such as sub-Saharan Africa, the Middle East, the Mediterranean, and India as heterozygosity for the mutation can provide protection [1,2]. Sickle cell patients present with several complications classified as acute and chronic. Acute complications include infections, severe anemia due to splenic sequestration, hyperhemolysis and aplastic crisis, and vaso-occlusion crises of different organs [3,4]. Chronic complications depend on the group of organs affected and may present with pain, cardiomyopathy, anemia, avascular necrosis, priapism, impaired kidney function, neurologic deficits, and strokes [5]. The survival of SCD patients depends mainly on their access to comprehensive care beginning with newborn screening and extending to managing severe complications. In 2014, Elmariah et al reported a median survival of 58 years for SCD and Sβ0 patients [6]. There are several modalities of treatment for SCD patients which include comprehensive care, medications such as hydroxyurea, L-glutamine, voxelotor, and crizanlizumab, blood transfusions whether it is simple or red cell exchange, curative hematopoietic stem cell transplant, and gene therapy. Given the risk of developing alloantibodies and/or autoantibodies with chronic blood transfusions in this category of patients due to exposure to different donor antigens, transfusion medicine services perform serological and molecular phenotyping of SCD patients and crossmatch units for every transfusion in order to reduce the risk of allo-immunization [7].

## Case presentation

A 19-year-old SCD patient with a previous history of sickle cell vaso-occlusion crisis and previous transfusions presented for red cell exchange. The patient’s blood type was determined as O-positive and the antibody screening test demonstrated a positive result in solid phase testing. Further characterization of the detected antibodies performed in the solid phase indicated reactivity consistent with anti-C (a clinically significant alloantibody to the big-C antigen in the Rh blood group system), anti-Jk(a) (a clinically significant alloantibody to the Jk(a) antigen in the Kidd blood group system), and warm autoantibodies. Notably, the patient tested serologically negative for the C (big-C) antigen but positive for the Jk(a) antigen. Further molecular characterization of the patient’s phenotype was performed by PreciseType™ HEA molecular BeadChip (Immucor Inc., Norcross, USA) with Basis 4G at a reference laboratory. The serological and molecular phenotypes are shown in Table 1. In addition, the patient was also found to have a single nucleotide variant (SNV) in the *GATA1* binding site in the promoter of the Duffy (Fy) genes. Hence there was no risk of forming anti-Fy (b) since the antigen is expressed on the endothelial surface.

**Table 1:** Serological and molecular phenotype of patient’s RBC

Blood Group	Antigen	Result (serology)	Result (molecular)
Rh Group	c (little-c)	Positive	Positive
	C	Negative	Negative
	e (little-e)	Positive	Positive
	E	Positive	Positive
	V	Not Tested	Positive
	Vs	Not Tested	Positive
Kell Group	K	Negative	Negative
	k (little-k)	Positive	Positive
	Kp(a)	Not Tested	Negative
	Kp(b)	Not Tested	Positive
	Js(a)	Not Tested	Negative
	Js(b)	Not Tested	Positive
Kidd	Jk(a)	Positive	Positive
	Jk(b)	Positive	Positive
Duffy	Fy(a)	Negative	Negative
	Fy(b)	Negative	Negative
MNS	M	Negative	Negative
	N	Positive	Positive
	S	Not Tested	Negative
	s (little-s)	Not Tested	Positive
	U	Not Tested	Positive

Given the results in Table 1, genotyping for Rh variants was performed at the same reference lab and resulted in probable RHD genotype: RHD*01, probable RHCE genotype: RHCE*cE/ RHCE*ceJAL. Accordingly, the patient's RHD/CE genotype predicted the following phenotype: D+, C+, E+, partial- e+, VS+(w/-), hrs+(w/-), hrb+(w/-), and JAL+. Further characterization of all SLC14AI(JK) coding exons (4 through 11) and their respective splice sites was performed at a reference lab and showed that an SNV (rs114641857) in intron 5 near exon 5 (c.470+12T/C) on chromosome 18. The patient had an exchange transfusion with R2/R2 ABO compatible units, negative for C (big-C) and Jk(a) antigens with extended phenotypic match, and achieved a hemoglobin S (HbS) level of less than 30%.

## Discussion

Red cell transfusion and exchange in SCD patients requires a careful approach and follow-up on patients to delay and prevent alloimmunization. Transfusion medicine services conduct thorough follow-ups on SCD patients by doing type and screen before each transfusion, and they establish serological and molecular profiles for each patient to select the safest units.

The Kidd (Jk) antigen is a 389 amino acid residue transmembrane glycoprotein. It is the red cell urea transporter and maintains the flow of urea into the red cells in order to stabilize the oncotic pressure across their membranes in addition to maintaining their shape [8].

The case we are presenting shows that the SLC14AI (JK) gene, on chromosome 18, had a single nucleotide variant (SNV, rs114641857) in intron 5 which could have resulted in aberrant splicing given its close location to exon 5. This SNV might be responsible for the lack of certain epitope/s that lead the immune system to alloimmunize and form anti-Jk(a) when units of Jk(a)-positive red cells are transfused. Such finding shows the importance of both serological and molecular testing, and the complementary role of all these tests to understand the whole immunological status of the patient. Although serological matching allows selecting units that prevent further alloimmunization, many antigens have mutations that can lead to partial expressions (missing certain epitope/s). Serologically, these mutated or partially expressed antigens lead to positive test results because the antibodies used in phenotyping target other epitopes that are present. However, the missing epitope can still be targeted by the immune system if positive red cell units (expressing all the epitopes) are transfused. Hence, type and screen as well as molecular testing in patients that are chronically transfused is important.

## Conclusions

Serological and molecular studies as well as type and screen testing are extremely crucial to map the antigens expressed on red cells for patients with SCD who require chronic transfusions or exchange especially when serologic discrepancy is detected between the phenotype and alloantibodies formed. Furthermore, further molecular studies and research are needed to map and understand the different mutations of red cell antigens and their clinical significance. For SCD patients, establishing a national database for each patient being chronically transfused or exchanged is crucial and may help to increase the safety margin of transfusions in this category of patients.
